# Three-Dimensional Face Recognition Using Solid Harmonic Wavelet Scattering and Homotopy Dictionary Learning

**DOI:** 10.3390/e24111646

**Published:** 2022-11-13

**Authors:** Yi He, Peng Cheng, Shanmin Yang, Jianwei Zhang

**Affiliations:** 1 National Key Laboratory of Fundamental Science on Synthetic Vision, Sichuan University, Chengdu 610065, China; 2 College of Computer Science, Sichuan University, Chengdu 610065, China; 3 School of Computer Science, Chengdu University of Information Technology, Chengdu 610065, China

**Keywords:** solid harmonic wavelets, scattering representation, 3D face recognition, sparse dictionary learning

## Abstract

Data representation has been one of the core topics in 3D graphics and pattern recognition in high-dimensional data. Although the high-resolution geometrical information of a physical object can be well preserved in the form of metrical data, e.g., point clouds/triangular meshes, from a regular data (e.g., image/audio) processing perspective, they also bring excessive noise in the course of feature abstraction and regression. For 3D face recognition, preceding attempts focus on treating the scan samples as signals laying on an underlying discrete surface (mesh) or morphable (statistic) models and by embedding auxiliary information, e.g., texture onto the regularized local planar structure to obtain a superior expressive performance to registration-based methods, but environmental variations such as posture/illumination will dissatisfy the integrity or uniform sampling condition, which holistic models generally rely on. In this paper, a geometric deep learning framework for face recognition is proposed, which merely requires the consumption of raw spatial coordinates. The non-uniformity and non-grid geometric transformations in the course of point cloud face scanning are mitigated by modeling each identity as a stochastic process. Individual face scans are considered realizations, yielding underlying inherent distributions under the appropriate assumption of ergodicity. To accomplish 3D facial recognition, we propose a windowed solid harmonic scattering transform on point cloud face scans to extract the invariant coefficients so that unrelated variations can be encoded into certain components of the scattering domain. With these constructions, a sparse learning network as the semi-supervised classification backbone network can work on reducing intraclass variability. Our framework obtained superior performance to current competing methods; without excluding any fragmentary or severely deformed samples, the rank-1 recognition rate (RR1) achieved was 99.84% on the Face Recognition Grand Challenge (FRGC) v2.0 dataset and 99.90% on the Bosphorus dataset.

## 1. Introduction

In recent years, point cloud and other metrical data have been utilized in multiple artificial intelligence applications; however, in the 3D face recognition research community, holistic-model-based methods still encounter issues: (1) there is a requirement of abundant training data to enable the capture of feasible features to form the large variations caused by the presence of rare and perturbing events, including occlusion/illumination/expression; (2) since the metrical data, e.g., raw point cloud, is generally sampled with structured high-resolution scanners with a restricted observing angle, some isometric deformations caused by exterior disturbances, e.g., pose variation or viewpoint variation, will inevitably confuse the inherent facial shape with sampling process noise and eventually, this issue will make raw point cloud representation behave like a non-uniform signal with dramatically varying intervals among points.

Solutions using the (semantic) model-matching process have achieved very high accuracy [1]; more recently, approaches using a 3D morphable model (3DMM) [2] have also achieved very high accuracy; however, the feasibility of key point detection and alignment relies on the constraints of large expression/pose variations.

**Figure 1:**
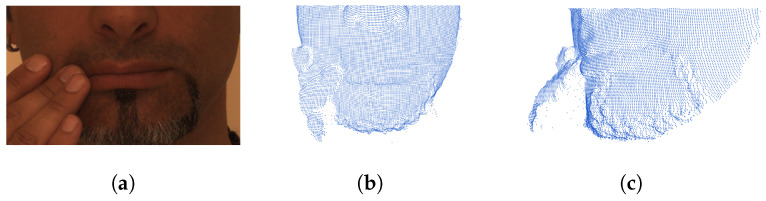
The different appearances of 2D and 3D representations: (**a**) the occlusion near the mouth in a raster image, (**b**) the same issue in a point cloud scan, (**c**) the second viewpoint of the occluded local area; the sample is from the Bosphorus dataset.

Approaches without key point detection utilize intermediate planar models such as depth image [1,3] to resample point clouds onto the regular domain and then apply 2D deep frameworks to extract salient features. However, these methods rely on extra information—including texture/RGB channels—to enhance discrimination, which tends to be more vulnerable/unpredictable and is suspected of wasting metrical resolutions. The study in [4] was an early trial of utilizing scattering representation to solve 3D face recognition problems. Note that a specific prepossessing procedure was applied to implicitly project raw point clouds into canonical multidirectional depth maps (e.g., X/Y/Z—normal map components used as independent input channels); this approach then learned the patterns of each component, respectively, with 2D scattering convolution and concatenated the filters’ responses as the global feature. This approach merely relied on geometric information to discriminate patterns from raw point clouds, but in more general scanning scenarios, complex and extrinsic disturbances, e.g., random 3D head poses/expressions/occlusions, may bring variations that affect both the signals on each predefined plane and the topology and geometry of the domain itself. For instance, the occlusion in a 2D raster image will result in some pop-up misplacements of the unrelated pixel values that may be highly diverged from the interested object, whereas in a 3D representation, the coarse shape frame is preserved with “additive” distortion/fragmentation in the local area (see Figure 1 for an illustration).

**Figure 2:**
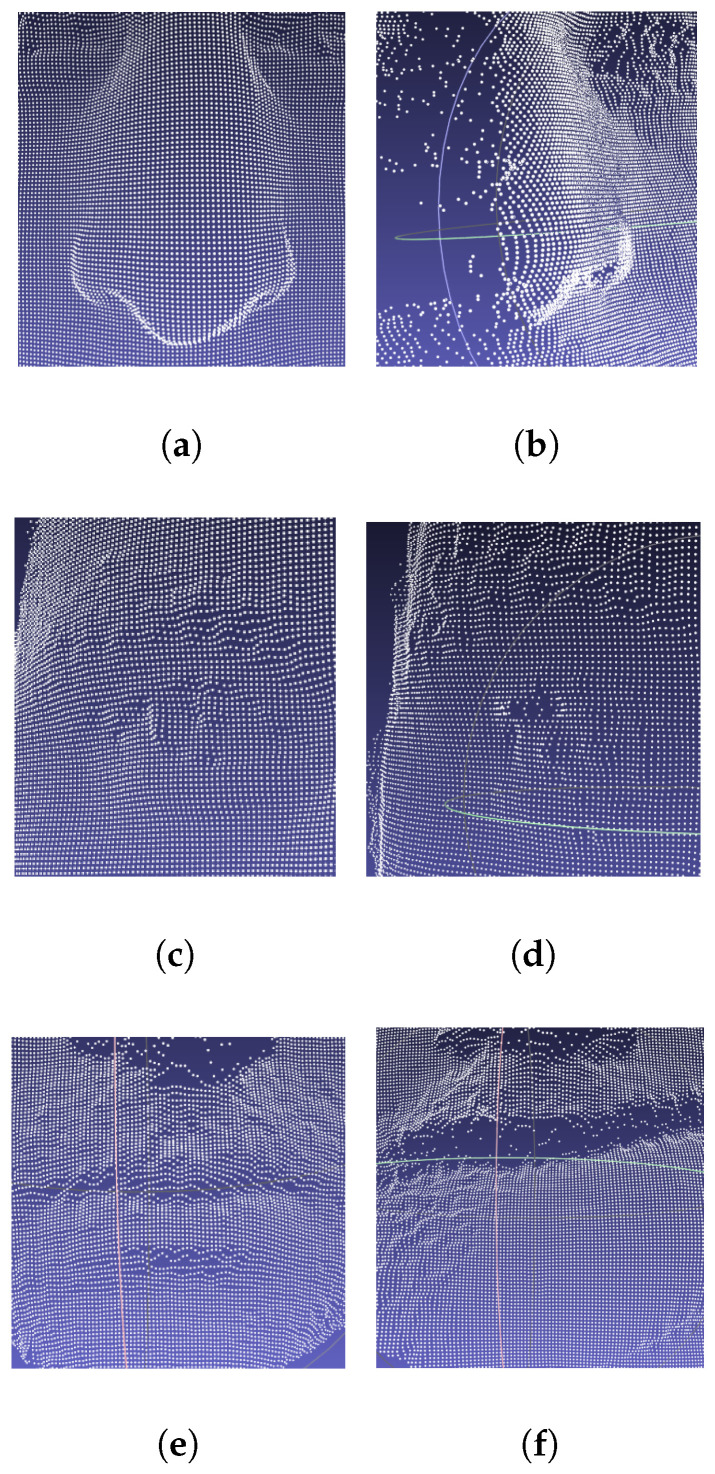
Low-fidelity local areas caused by pose variance/expression/occlusion. Top: (**a**) frontal; (**b**) posture (45${}^{\circ }$). Middle: (**c**) neutral; (**d**) surprised. Bottom: (**e**) non-occlusion; (**f**) occlusion.

The benefit of geometric representation is that the integrity of the underlying structure can be expected and novel issues can be acting in multiple forms, as illustrated in Figure 2, where we picked one identity from the Bosphorus dataset with its coupled local variants. The first column shows the nose area in different (sampling) pose angles; it is clear that the integrity has been demolished and the frequency has been shifted (e.g., the nose tip area). Similar randomness emerged in the intrinsic deformations from expressions/occlusions, as demonstrated in the second column (the same subject’s right eye area, with a neutral expression at the top and a surprised expression at the bottom) and the third column, where the mouth area was rendered both with and without occlusions. The above-mentioned methods, in this case, can spoil the surface assumption of faces, which leads to over-smoothed representations.

The last element that degrades the performance of the above-mentioned regular domain-based method is related to non-uniform sampling in relaxed scenarios, as illustrated in Figure 2, which shows that the extrinsic deformation also causes complicated local frequency shifting. As a result, the discriminative feature for recognition becomes crumpled into a wide frequency range. If we directly apply the (statistical) model-based methods (e.g., 3DMM) and compute the integration of the (dis)similarity measure along the predefined vertex paths or even along the regular domains (e.g., depth map), without the indispensability of homogeneity or the stability of such underlying geometry, the discrimination will probably become lost; this is because such predefined metrics will probably regress to represent identity-unrelated properties such as (spectral) energy. Otherwise, if we eliminate these high-frequency variances through holistic denoising methods, the inter-subject dissimilarity will tend to be blurry since every face has an approximately consistent sketch shape.

We observed that the raw face scan samples naturally contained multiple species of noise/perturbations relating to sampling processes and other stochastic behaviors. Furthermore, by considering different facial identities as a family of stochastic processes, the identity-related properties were considered the underlying processes that merely related to the neutral and canonical appearance of the characterized physical face. In this setting, point cloud face recognition is similar to high-resolution texture discrimination, where Julesz gave the classical textons hypothesis [5,6]: by defining a finite collection of realizations of a texture, the statistical difference underlies preattentive discrimination. Accordingly, if we construct global measurements with desired invariants of unwanted variables, e.g., head poses/expressions, then the identity can be restored by comparing the “3D textons” [7] of a high-resolution human face (scan).

Through such intuition, face scans have several significant differences: (1) point clouds have no inherent correlation to the illumination/texture material, which shows the potential to directly obtain identity information merely from their geometry; however, the illumination/expression variations will lead to local deformations and (2) point clouds have no regular underlying cardinality, which disables the convolution-like operations and makes them an under-defined representation, so the results using convolution will be rather unstable with the order permutation. Therefore, we searched for a stable representation against global rotations/order permutation and spatial translations that should still be capable of encoding sufficient high-resolution geometry; however, if accompanied by similar dictionary learning to find the co-occurrences of filter responses across diverged illumination and pose conditions, a sparser feature can be restored from such redundant representations.

To accomplish the above configuration, we first needed an alternative descriptor to capture features at the local scale. The micro-structures lie in scattered point sets so the representation should be regularized into a more canonical manner and the non-uniformness, e.g., frequency shifting, should be mitigated with a stabilizing operation.

Secondly, to build the 3D texton vocabulary (or more specifically, dictionary) with limited observations, we needed an efficient framework with the potential to disentangle variations according to an induced operational path, where certain desired invariance, stability, and consistency properties can be expected. Specifically, we proposed the use of a solid harmonic scattering transform [8] as a stabilizer and a local reference, lattice-based density estimation descriptor to capture such features for raw point cloud face recognition. Furthermore, we implemented a sparse scattering deep convolutional neural network based on [9] to build a local dictionary of the microstructures of a human face.

**Figure 3:**
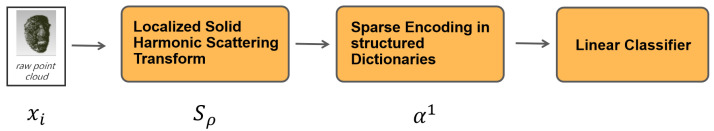
The sparse dictionary learning framework for 3D face recognition: the original solid harmonic wavelet scattering was adapted to piece-wise operations; with the sparse approximation coding from a union of learned dictionaries, the irregular raw point cloud faces can be classified merely through shapes.

The proposed approach is illustrated in Figure 3.

In our approach, the raw point cloud faces are reorganized and down-sampled first with a generic resampling method such as farthest point sampling (FPS) [10]; then, one must consider the down-sampled points as entries to compute the nearest neighbors as the original local spatial signals. After having such subsets, we apply a reference lattice that originates at each entry point with a bump Gaussian function to estimate the probability of the appearance of a sampled point at such canonical grid-like positions and imitate a continuous function. After that, we apply a solid harmonic scattering transform to each point subset and obtain the representation of the 3D translation and rotation invariant scattering coefficients. With this stable representation, we then apply an iterative sparse thresholding coding (ISTC) [9] block to learn a discriminative feature space that reduces intraclass variability while preserving class separation through projections over unions of linear spaces.

Our major contributions are summarized as follows:

- Inspired by the high-resolution texture discrimination, this paper proposes a novel process–model approach to obtain the discriminative and stable facial features from pure point coordination representation for automatic face recognition; here, the facial shape clues are enhanced in a regularized domain.
- We modify the original holistic solid harmonic wavelet scattering transform approach into a windowed integral function to provide a higher-resolution representation.
- We learned a 3D facial texton dictionary, which is specifically based on the co-occurrences of filter responses across different extrinsic perturbances, e.g., head pose/illumination variation/occlusion, and succeed in achieving competitive recognition accuracy compared with alternative currently available methods.

This paper is organized as follows. In Section 2, we review the related works that have been published in recent years. In Section 3, we briefly recall solid harmonic scattering and sparse dictionary learning and then present our spatial construction for extracting the localized features of point cloud faces. The experimental results are stated in Section 4 and we conclude the paper in Section 5.

## 2. Related Works

From scene modeling to molecular imaging, metrical data such as point cloud/mesh data have been applied to the representation of multiple scaling geometries/structures of multitudinous objects or abstract concepts (e.g., graphs/manifolds), and the fast-growing field of geometric deep learning [11] has spawned multiple promising offspring; among these, here, we solely focus on those that directly utilize unstructured point cloud data.

### 2.1. Point Cloud Deep Learning

As immediate metrical results from the sensors, the points in the representations can carry intrinsic features in their natural manner, whereas regular, grid-like imaging sampling leads to the inevitable entanglement of environmental variants and objective signals; hence, this requires object detection/background segmentation, lengthy supervised feature abstraction, or data argumentation. The approach to consuming raw point clouds starts by resampling the points into continuous spatial voxels with statistic features that tend to lose higher-frequency components [12]; otherwise, an adapted multiple scaling strategy would be needed, which would require an excessive computation cost to cover the varying spectrum because of the sparsity and non-regularity of the general point cloud representation [13].

Methods for extracting order-invariant features on point clouds were discussed for Pointnet [14] and Pointnet++ [15], which provide point-wise feature and hierarchical representations. The authors of [2] utilized this idea to solve point cloud face classification; however, this approach requires the learning of a Gaussian process morphable model [16] to encode the holistic features of real face samples to mitigate the intraclass variances from the face-scan phase.

Alternative improvements involve the construction of more flexible underlying affinity structures and learning features through them; multiple challenging problems in metrical data have been solved—with satisfying results—using these techniques, including semantic scene segmentation/3D object classification [14,17,18].

### 2.2. Dictionary Learning on Scattering Coefficients

A scattering transform has the ability to mitigate undesired group-structured operations in well-defined domains, e.g., image/voice signals, with predetermined wavelet filter banks [19,20,21]. Predefined harmonic wavelets can bring translation–rotation invariances and linearize isometric deformations and have been utilized as a superior tool for representing molecules’ fine 3D geometry, namely the solid harmonic wavelet scattering transform [8].

However, unlike atoms’ orbital positional distributions, a raw face point cloud yields a coarse underlying smooth surface but behaves with non-guaranteed differentiability. The Euclidean learning methods would probably fail to converge. The authors of [22] proposed an approach for the overlap of multiple smoothed position signals, allowing for the representation of periodic structures. The idea of combining a scattering transform approach with a deep network has also been developed [23,24]; additionally, a recent attempt at solving complicated classification problems with scattering representation achieved a remarkably fast-converging performance with potential in mathematical analytic implementations [9]. Furthermore, their work relies on a classical, active technology—sparse dictionary learning [25]. We utilized this idea to select discriminative signal components to prevent our model from regressing to a representation of irrelevant deformations.

### 2.3. 3D Face Recognition

As discussed in [2,26], research on the 3D facial recognition problem has developed in several major directions: (local) feature-based, (holistic) model-based, and matching-based methods. From a general perspective, we can see an underlying trend of reducing the necessity of the registration phase/domain matching—on account of the development of acquisition techniques that enforce more regular raw scanning results—while stronger computation methods and facilities progressively enable parallel processing on high-throughput data streams. As a characteristic indicator method, the iterative closest point (ICP) [27] matching scheme played a significant role in [28,29,30,31], where the above isometric deformations have been eliminated by spatially aligning faces into a common direction.

Holistic methods have been developed in recent years to reduce the necessity of registration; for example, [32] proposed the Markov random field (MRF)-model-based approach to select discriminant features on posterior marginal probabilities. Later works, such as [1,3], concentrated on adapting a 2D learning framework for 3D scans, where a deep range image has commonly been utilized for medium representation, which might partially ignore the indifferentiability in spaces, e.g., 3D rotation by mass-supervised training. The authors of [2] provided an a priori model-based argumentation strategy to avoid the above-described question.

Owing to space constraints, we only named a few of the relevant works that studied the 3D face recognition problem; as we stated, a study on the construction of more intrinsic representations for facial recognition is necessary.

## 3. Materials and Methods

In this section, we first present our approach to modeling identities under noisy environments and a method for constructing stable representations; additionally, we describe the sparse dictionary learning structure for feature selection. Finally, we present our overall framework for effective jointly learning discriminative representations.

### 3.1. The Stochastic Process Model on Point Cloud Faces

Let one identity be denoted as $X_{i}$, with *i* ranging through the different identities; we consider point cloud faces as realizations of a modeled noisy observation process as follows:

(1) $$x_{i}\in L_{\theta }\left(X_{i}\right)$$

where each sample only provides spatial coordinates and can be expressed as a vector $x_{i}=\left\{r_{k}\in R^{3}:k=1\ldots K\right\}$, where *K* is usually in 10k magnitudes. The $L_{\theta }$ is a function that models the above geometric transformation, illumination, and occlusion variances. In addition, the θ can be seen as a low-dimension random vector encoding the global illumination and rigid affine transformations [33], though in general, the ergodicity of $L_{\theta }$ is hard to be satisfied.

As a possible solution, a graph-based method [14] applied a dynamic approximation procession to transform raw point clouds into uniform point/vertex sequences with lengths of thousands; then, it was used to compute the corresponding embedded features in order to imitate a universal characteristic representation.

This method shed light on defining signals with near-independent distributions between global and local variables; however, it required heavy training to realize the asymptotic stability, which is neither available nor necessary in modeling more consistent structures, e.g., faces, where geometric deformation and/or illumination/pose variances will not lead to large universal interferences.

Upon these observations, we built Euclidean lattices and learned the spatially aware features using solid scattering transform-based local operators; by applying subsequent sparse dictionary learning in the scattering domain, the uncorrelated signal components of the identity in question were jointly learned and inhibited.

**The local 3D lattice operator:** We used $p_{0}$ to denote the centroid of $x_{i}$, which was easy to obtain, and from $p_{0}$ we constructed the 3D global coordination, $M_{xyz}$. The succeeding step used farthest point (FP) [10] sampling on $x_{i}$; note that we only needed to draw the countable C<200 points as query points, $P_{0}={\left\{p_{c}\right\}}_{c⩽C}$, for the subsequent spatial thresholding nearest-neighbor searching. From each $p_{c}$, we drew the N nearest points from their ambient spaces to form a leaf subset:

(2) $$P_{c}={\left\{r_{c,n}\in N_{R_{c}}\left(p_{c}\right):diam\left(p_{c}\right)<R_{c}\right\}}_{n⩽N}$$

where we picked a threshold radius—$R_{c}=\min∥p_{c}-P_{0}/\left\{p_{c}\right\}∥$—to dynamically assure coverage. Furthermore, within each ambient space, we associated a 3D local lattice coordination $\mu_{xyz}\subset M_{xyz}$ and defined the overall density estimation function as the concatenation of C local areas:

(3) $${\hat{\rho }}_{\Omega }\left(\mu \right)=\left({\hat{\rho }}_{1},\ldots ,{\hat{\rho }}_{c},\ldots ,{\hat{\rho }}_{C}\right)$$

where each ${\hat{\rho }}_{c}$ was parameterized by

(4) $${\hat{\rho }}_{c}\left(\mu \right)=\sum_{n=1}^{N}G\left(\mu -r_{c,n}\right)$$

which is a sum of the Gaussian densities centered on each $r_{c,n}$. This spatial construction sliced each $x_{i}$ into *C* local receptive fields; we adjusted the width parameter, σ, of the Gaussian equivalent to the distance from the nearest alternative entry point, i.e., $\sigma_{c}\to \sup\left(∥r_{c}-{r_{c}}^{\prime }∥\right)\forall r\in N_{c}$. By renominating each $\rho_{c}$ with indicator function $I_{\rho_{c}}$, a raw point was transformed into a naive Borel set, which had a uniform probability measure, so we defined the global piece-wise density function as $\rho_{\Omega }=⋃\rho_{c⩽C}$.

**Figure 4:**
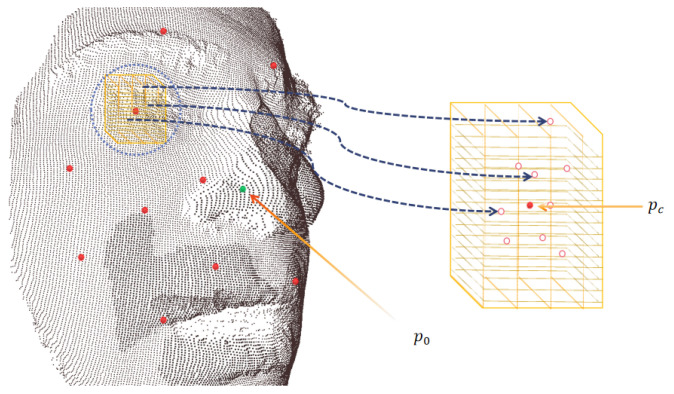
**Left**: The sparse entry points (in red) obtained by the FPS algorithm. **Right**: The local 3D lattice patch operators used to cover the ambient spaces around the entry points.

This approach encoded a raw point cloud face into a more regular continuous probability density representation, with local fields being invariant to the permutations of the input order; each characteristic vector also had a corresponding length, which enabled the windowed operations (See Figure 4 for illustration).

However, the above isometric deformations not only broke the order consistency but also gave rise to mixed deformations and polluted the geometric features; therefore, we needed to add a stabilizer to obtain the rotation and translation invariances.

To illustrate the operation, a piece of pseudocode is given below as Algorithm 1:
**Algorithm 1** The Local Lattice Operation:**Require**: $p_{0}=\left(x_{0},y_{0},z_{0}\right)$ (the centroid of a raw face scan $x_{i}$), $x_{i}=\left\{r_{k}\in R^{3}:k=1\ldots K\right\}$1:**Set**$p_{0}$ as the initial point of farthest point sampling and2:**Draw***C* points ${\left\{p_{c}\right\}}_{c⩽C}$ from $x_{i}$3:**for**$p_{c}$ in ${\left\{p_{c}\right\}}_{c⩽C}$ **do**4:   **Set** $p_{c}$ as the origin, and5:   Compute $P_{c}=KNN\left(p_{c},N\right)$6:   Compute local lattice $\mu =\left\{m\cdot dx,n\cdot dy,o\cdot dz\right\}$7:   Compute local density estimation ${\hat{\rho }}_{c}\leftarrow \sum_{n=1}^{N}G\left(\mu -r_{c,n}\right)$8:**end for**9:Concatenate local densities to form the overall function as${\hat{\rho }}_{\Omega }\left(\mu \right)=\left({\hat{\rho }}_{1},\ldots ,{\hat{\rho }}_{c},\ldots ,{\hat{\rho }}_{C}\right)$10:**return**${\hat{\rho }}_{\Omega }$

**Figure 5:**
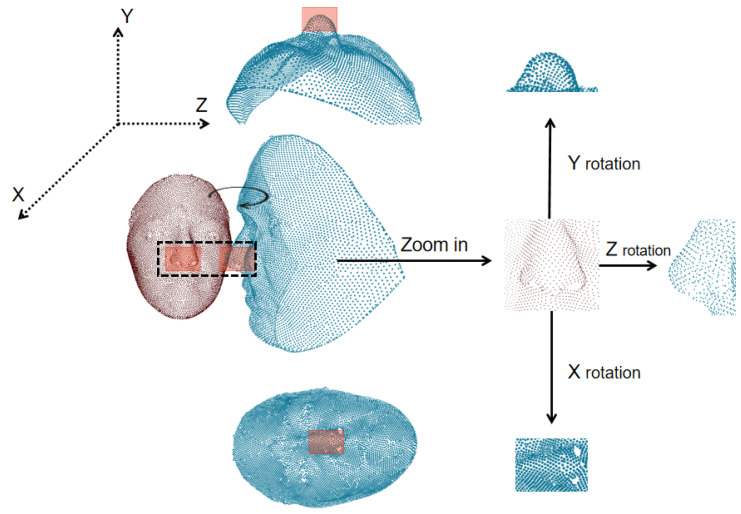
The rigid rotation caused by pose variations; this figure merely gives several discretized realizations. The point subsets in blue indicate the rotated version of the original local areas around the nose tip.

Note that since the direction of local lattice μ was exactly covariant to the global coordination of the scanned face, the above-obtained local density feature actually exposed itself to a risk of being sensitive to rigid rotation, as well as to the order permutation of the grid position points (see Figure 5 for an illustration). Therefore, we needed to construct a stabilizer to eliminate such isometry.

**Windowed solid harmonic wavelet scattering:** A scattering transform is a geometric deep learning framework that replaces learning-based cross-correlation kernels with predefined filter banks. Induced stability for multiple species of isometrics and translation invariances can be prescribed with a group-invariant structure built from a deliberately configured wavelet filter bank [19,20]. For 2D signals (e.g., images), the constitutive substructure in a scattering network comprises the wavelet filters with zero integrals and yields fast decay along $∥\mu ∥$; each can be parameterized by a rotation parameter, θ, and dilation parameter, *j*, as

(5) $$\psi_{j,\theta }\left(\mu \right)=2^{-2j}\psi \left(2^{-j}r_{-\theta }\mu \right)$$

where r∈G belongs to a finite-rotation group of $R^{d}$.

For 3D signals—as in 3D face recognition with point cloud samples—3D rotation invariance is crucial since the random pose variation may provide an alias for the local density feature obtained by our local lattice operator (see Figure 5). Accordingly, we built a stabilizer in the solid harmonic scattering approach from [8], whereby solving the Laplacian equation with the 3D spherical coordinates and replacing the exponent term in the spherical harmonic function, $Y_{\ell }^{m}$, the solid harmonic wavelet can be expressed as follows:

(6) $$\psi_{\ell ,m}\left(r,\theta ,\varphi \right)=\frac{1}{\sqrt{{\left(2\pi \right)}^{3}}}e^{-1/2r^{2}}r^{\ell }Y_{\ell }^{m}\left(\theta ,\varphi \right)$$

In addition, by summing up the energies over *m*, a 3D covariant modulus operator can be defined as

(7) $$U\left[j,\ell \right]\rho \left(\mu \right)=(\sum_{m=-\ell }^{\ell }|\rho 🟉\psi_{\ell ,m,j}\left(\mu \right){|}^{2}{)}^{1/2}$$

**Figure 6:**
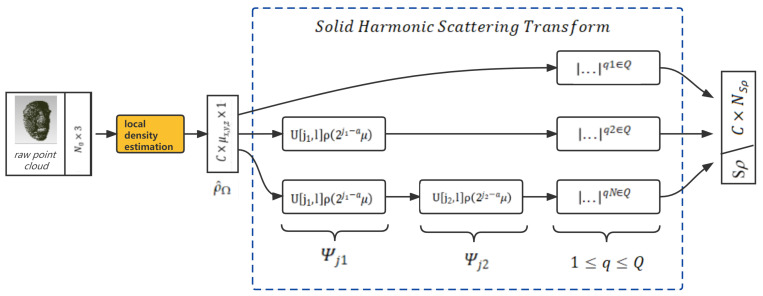
Illustration of the localized solid harmonic scattering transformation; the dashed blocks represent the extracted invariant representations from each local ambient space.

In short, a solid harmonic scattering transform is defined as the operation of summing up the above modulus coefficients over μ to produce translation-/rotation-invariant representation within each local field (see Figure 6 for illustration). Furthermore, by raising Ux[j,ℓ]ρ(μ) to exponent q and then sub-sampling μ at $2^{j-\alpha }$ with an oversampling factor—α=1 to avoid aliasing, the first-order solid scattering coefficients are

(8) $$S\left[j_{1},\ell ,q\right]\rho =\sum_{\mu }{\left|U\left[j_{1},\ell \right]\rho \left(2^{j_{1}-\alpha }\mu \right)\right|}^{q}$$

Then, by iterating subsampling at intervals $2^{j_{2}-\alpha }$ with $j_{2}>j_{1}$ and recomputing the scattering coefficient on the first-order output, we obtained the following second-order scattering transform:

(9) $$S\left[j_{1},j_{2,}\ell ,q\right]\rho =\sum_{\mu }{\left|U\left[j_{2},\ell \right]U\left[j_{1},\ell \right]\rho \left(2^{j_{2}-\alpha }\mu \right)\right|}^{q}$$

These representations can hold local invariant spatial information up to a predefined scale, $2^{J}$; in our case, this was adjusted to be equivalent to the local threshold diameter, $N_{R_{c}}$. Furthermore, we needed to extend this operation to a universal representation. Here, we defined the windowed first and second solid harmonic wavelet scattering as follows:

(10) $$S_{\cup }\left[j_{1},\ell ,q\right]\rho_{\Omega }=\overset{C}{\underset{c=1}{⋃}}|U\left[j_{1},\ell \right]\rho_{c}{|}^{q}$$

(11) $$S_{\cup }\left[j_{1},j_{2},\ell ,q\right]\rho \overset{C}{\underset{c=1}{⋃}}|U\left[j_{1},\ell \right]U\left[j_{2,}\ell \right]\rho_{c}{|}^{q}$$

For a better illustration, a brief pseudo-code is stated below as Algorithm 2:   
**Algorithm 2** Windowed Solid Harmonic Wavelet Scattering**Require:** ${\hat{\rho }}_{\Omega }$ (local density features), *J* (scale parameter), *L* (rotation phase parameter), *Q* (exponential parameter)1:**Set** wavelet t$\psi_{\ell ,m}\left(r,\theta ,\varphi \right)$ according to a predefined parameter (J,L) with Equation [6]2:**for**$\rho_{c}$ in ${\left\{\rho_{c}\right\}}_{c⩽C}$ **do**3:   **for** 0⩽j⩽J **do**4:     Compute the dilated modulus operation on scattering convolution $\rho_{c}🟉\psi_{\ell ,m,j}\left(\mu \right)$, e.g., Equation [7]5:   **end for**6:   Compute the first-order coefficients $S[j_{1},\ell ,q]\rho$ as Equation [8]7:   Compute the second-order coefficients $S[j_{1},j_{2},\ell ,q]\rho$ as Equation [9]8:   Concatenate first and second coefficients as the local invariant representation $S_{\rho_{c}}$9:**end for**10:Concatenate ${\left\{S_{\rho_{c}}\right\}}_{c⩽C}$ as the global invariant representation $S_{\rho_{\Omega }}$11:**return** $S_{\rho_{\Omega }}$

### 3.2. Piece-Wise Smoothed Solid Harmonic Scattering Coefficient Representation

The above strategy makes the representation stable to local deformations, and since face point clouds share a largely consistent global structure, it allows us to represent them even if no effective global embedding exists.

To balance the computation complexity and resolution in our experiments, we chose C=128, J=7, and q∈Q={1/2,1,2,3}; here, the above windowed operation and scattering coefficients were implemented with the Kymatio software package [34]. To simplify our notation, we wrote the scattering representation in shorthand as follows:

(12) $$S\rho_{c}={\left\{S\left[p\right]\rho_{c}\right\}}_{p}$$

where *p* is the union of the first and second indices $\{\left(j_{1},\ell ,q\right)$ and $\left(j_{1},j_{2},\ell ,q)\right\}$, respectively, and the overall scattering coefficients of a point cloud face are

(13) $$S\rho_{\Omega }={{\left\{S\rho_{c}\right\}}_{c}}_{⩽C}$$

**Figure 7:**
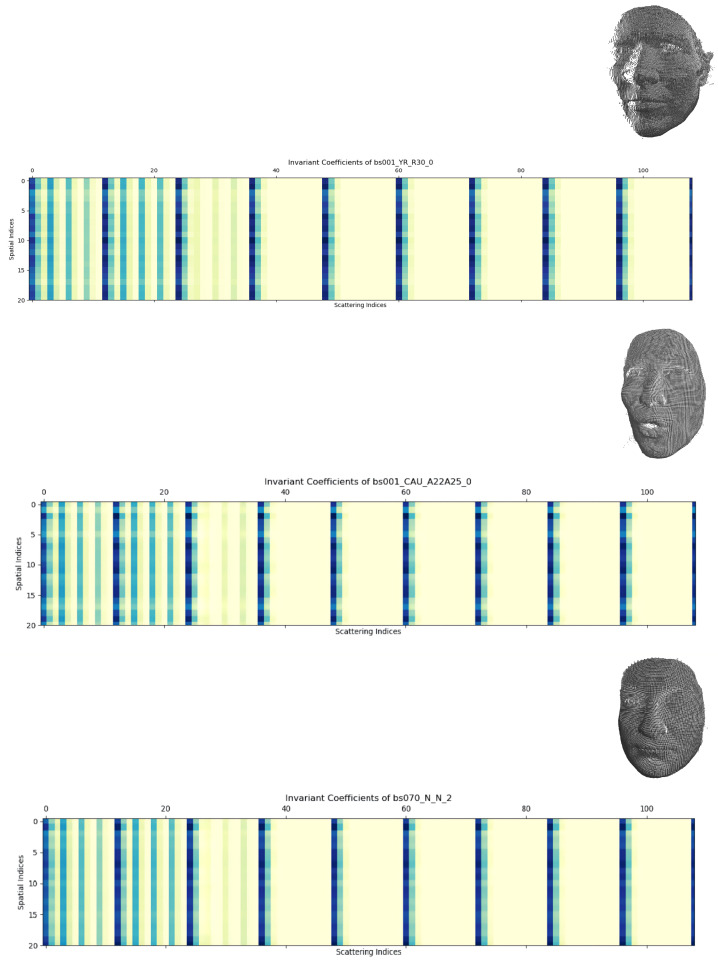
Scattering invariant coefficient representations: top—identity bs001 from Bosphorus with a $30^{\circ }$ yaw rotation; middle—bs001 with an action unit combination (expression); bottom—bs070 with a neutral frontal position.

To give an illustration of this representation, we mapped the first-order scattering coefficients of two identities (bs001 and bs070) onto the scattering indices shown in Figure 7; identity bs001 from Bosphorus had two realizations and we could see that, although there was a significant visual difference between them, their scattering coefficients had a similar appearance.

To enhance the discrimination of this representation—and inspired by [7]—in the next section, we construct a “facial scattering coefficients dictionary” from the above representation to associate multiscale properties for 3D facial recognition. Specifically, based on the good results from the 2D scattering coefficients in [9], we follow their idea for utilizing supervised dictionary learning to select the most relative classification features from the 3D scattering coefficients.

### 3.3. Constructing a Local Dictionary with Semi-Supervised Sparse Coding on Scattering Coefficients

The scattering representation brings desired properties including Lipschitz continuity to translations and deformations [19]; however, the overall structure constructed using FPS and local nearest searching and normalization assumes uniform energy distribution among the realizations of each identity. In real scan scenarios, this assumption can be damaged when permutations, e.g., occlusion/rigid overall rotation, break the integrity of the face samples. In some severe cases, a certain portion of points are missing from face samples in Bosphorus. To reduce this category of intraclass variance, we imposed the homotopy dictionary learning framework presented by [9] to build a local coefficient dictionary. Then, we trained the network to select the most relative classification features from the scattering coefficients.

**Supervised Sparse Dictionary Learning:** The idea of selecting the sparse combination of functions from redundant/over-complete dictionaries to match the random signal structure was presented by [35] and flourishes in multiple fields related to signal processing. Supervised dictionary learning was first presented by [36] to solve the following optimization problem:

(14) $$\min_{D,\Theta }\sum_{j}\ell (x_{j},y_{j},\Theta ,\alpha^{*}\left(x_{j},D\right))$$

where Θ indicates a simple classifier’s parameters; *ℓ* is the loss function for computing the penalization on the prediction $\left(x_{j},y_{j}\right)$; $\alpha^{*}$ is the sparse code of the input signal, $x_{j}$, with the learned dictionary, *D*.

In our problem, the input signal, $S\rho_{\Omega }$, had a union form; hence, we constructed a global dictionary with structured local dictionaries defined as:

(15) $$D\;=\;\left[D_{1},...D_{c}...,D_{C}\right]$$

where ${\left\{D_{c}\right\}}_{c=1}^{C}$ are C sub-dictionaries with a certain structure—$D\in R^{K\times C\times N}$. Here, K indicates the length of the local pseudo coordination, *p*, of $S\rho_{\Omega }$; the aim was to represent *B* input samples (*B*—batch size) as linear combinations of *D*’s elements. Each $D_{c}$ had N=512 normalized atoms/columns—${\left\{d_{n}\right\}}_{n=1}^{N}\in R^{K}$. Then, the sparse approximation optimization was used to solve

(16) $$\underset{D,\alpha_{i}}{\arg\min}\sum_{i=1}^{B}{∥S\rho_{i}-D\alpha_{i}∥}_{2}^{2}+\lambda_{*}{∥\alpha_{i}∥}_{0}$$

where $\alpha_{i}$ is the concatenated sparse codes $\alpha_{i}=\left[\alpha_{i,1},...\alpha_{i,c}...,\alpha_{i,C}\right]$. Suppose the optimized sparse coefficient matrix is $A\in R^{K\times N\times C\times B}$ for a batch of input signals, ${\left\{S\rho_{i}\right\}}_{i=1}^{B}\in R^{K\times C\times B}$, where each sub-dictionary has a local code $\alpha_{c}\in R^{N\times B}$.

**Expected Scattering Operation:** Since we regrouped the raw point clouds and individually computed the invariant representations, $S\rho_{c}$, the windowed representation also had a non-expansive property; within each local field, the translation converged to being negligible by taking J→∞.

In practice, this will possibly bring ambiguity. By setting a small *C*, each field becomes too large and results in the loss of higher-frequency components. Yet, for a larger *C*, the computing complexity amounts to O(CS), and the optimization of such a concatenation will lead to supernumerary consideration, e.g., vanishing gradients.

Thanks to the integral normalized scattering transform [20], which preserves a uniform norm by utilizing the non-expansive operator ${\bar{S}}_{{}_{C}}$, we considered our question of structured learning for some random processes using the supposed condition. For the underlying distributions of point cloud faces yet to be established in practice, we focused on finding a solution with the above intuition and incautiously assumed our representation to be a stationary process up to negligible higher components; thus, the metric among (a batch of) spatial realizations reduced to a summation of the mean-square distances is

(17) $$\Delta \left(S\rho -DA\right):=\frac{1}{BC}\sum_{i}^{B}\sum_{c}^{C}{∥S\rho_{i,c}-D_{c}\alpha_{i,c}∥}_{2}^{2}$$

This definition is simple but effective as a regression term with a forward–backward approximation, which is based on an operation called proximal projection [37].

(18) $$\begin{array}{c} A^{*}=prox_{\lambda }\left(S\rho \right)\forall \left(S\rho ,A\right)\in R^{N=KCB}\times R^{N=KCB}\\ \Leftrightarrow A-S\rho =\Delta (S\rho -DA) \end{array}$$

where it encloses a solution with a forward step by computing a reconstructed $\tilde{S}\rho$ and a backward step by putting it back into the proximal projection operator, updating λ and *D*. Since our aim was to implement an efficient classification model, the sparse code should be able to preserve the principle components of the input signal; additionally, with the experimental observation of the point cloud faces’ solid scattering coefficients, we saw most energy being carried by its rare lower-frequency components and characterized by larger-magnitude coefficients; therefore, we picked the recent generalized ISTC algorithm [9], which adopts an auxiliary dictionary to accelerate convergence. Here, the ReLU function acts as a positive soft thresholding implementation of proximal projection. Then, the optimization can be reached in an unsupervised n⩽N-iteration-updating scheme, expressed as follows:

(19) $$\alpha_{n}=\mathrm{ReLU}\left(\alpha_{n-1}+D^{T}\left(\beta -D\alpha_{n-1}\right)-\lambda_{n}\right)\;\mathrm{for}\;n⩽N$$

**Figure 8:**
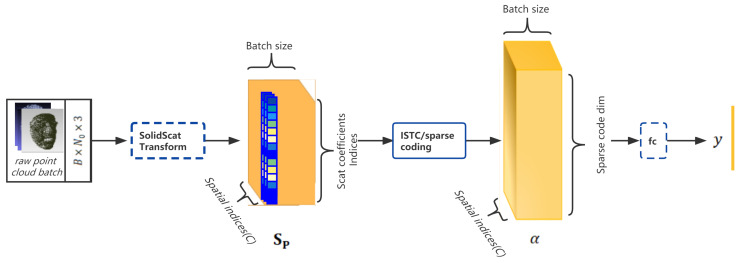
The architecture of the pure-spatial-coordination-based framework for 3D face recognition.

The overall architecture is shown in Figure 8.

To effectively demonstrate our methods, a pseudo-code is given in Algorithm 3:
**Algorithm 3** Dictionary learning on local facial coefficients**Require:** {(y,x)} (training set), *D* (initial dictionary), $\lambda_{1}$ (initial Lagrange multiplier, e.g., thresholding bias), θ (classifier parameter), *N* (number of iterations), τ (learning rate), *v* (regulation parameter)1:**Draw** a batch of samples ${\left\{\left(y_{j},x_{j}\right)\right\}}_{j⩽Batchsize}$ and compute the scattering coefficients using the methods in Section 3.2; denote the coefficient vectors as ${\left\{\beta_{j}\right\}}_{j⩽Batchsize}={\left\{S_{\rho_{\Omega }}\right\}}_{j⩽Batchsize}$2:**for**1⩽j⩽Batchsize**do**3:   **for** 1⩽n⩽N **do**4:     Compute $\alpha_{n}=\alpha_{n-1}+D^{T}\left(\beta_{i}-D\alpha_{n-1}\right)-\lambda_{n}$ where $\alpha_{0}=0$5:     Compute $\alpha_{n}^{🟉}=ReLU_{\lambda_{n}}\left(\alpha_{n}\right)$6:     **Update** $\lambda_{n}=\lambda_{max}{\left(\frac{\lambda_{max}}{\lambda_{🟉}}\right)}^{-n/N}$7:   **end for**8:   Compute $\lambda_{N}=\lambda_{🟉}$ and $\alpha_{N}=ReLU_{\lambda_{N}}\left(\alpha_{N-1}\right)$9:**end for**10:Compute the classification loss $\sum_{j}^{}Loss\left(D,\lambda_{N},\theta ,\beta_{j},y_{j}\right)$11:Update the parameters by a projected gradient step [36]12:$\theta \leftarrow \prod_{\theta }^{}\left[\theta -\tau (\nabla_{\theta }Loss\left(D,\lambda_{N},\theta ,\beta_{j},y_{j}\right)+v\theta \right]$,13:$D\leftarrow \prod_{D}^{}\left[D-\tau (-W^{T}D\alpha_{N}+\Delta \left(S\rho -DA\right)\right]$14:**return** Learned Dictionary *D*

## 4. Results

The goal of this paper was to construct a geometric deep face recognition learning model that relies merely on geometrical features and eliminates complicated embedding procedures, as well as hand-crafted feature alignment. We compared the performance of our approach with those of relevant methods using two well-known datasets—Bosphorus [38] and Face Recognition Grand Challenge v2.0 [39]. We found that our structure was more analytic and obtained competitive results. In Section 4.1, we describe our evaluation protocol and metrics, and in Section 4.2, we detail our implementation and present some parameter analyses. In Section 4.3, we demonstrate the parameter tuning process. The main results are presented in Section 4.4.

### 4.1. The Evaluation Protocol and Metrics

We applied the general evaluation protocol by comparing the rank-1 recognition rate (RR1) and the verification rate (VR) with the false-accept/positive rate (FAR/FPR) = $1\times 10^{-3}$ as the key performance metric. We compared our method with other competing methods, where the rank-1 recognition rate (RR1) was defined as the proportion of positive label predictions out of the total number of predictions for the whole test set. The total number of label predictions consisted of the sum of the positive and negative predictions. By further clarifying the positive results into the true-positive rate (TPR) and the false-accept/positive rate (FAR/FPR), the verification rate was defined as the portion of positive results under a certain false-accept/positive rate (FAR/FPR).

### 4.2. Implementations

The backbone of our implementation was based upon the work of [9], who achieved competitive results in 2D image classification problems with a mathematical analytical structure. In our study, the data structure had prominent differences; therefore, we needed to perform modifications, as follows.

(1) The 3D solid scattering coefficient representation: As introduced in Section 2, we transformed the raw point cloud into representative zero-, first-, and second-order cascades of the solid harmonic scattering coefficients (shown in Figure 5); the implementation was based on an open-source framework [34].

The typical size of a sample in the Bosphorus and FRGCv2 datasets ranges from 30 k to 100 k; as discussed in Section 3.1, a sufficient *C* of partitions can reduce the error in estimating the dimensional metric; however, a rising *C* requires increasing arbitrary coefficients, and we needed to balance the computation load between requesting the fineness of the representation and processing to obtain high-efficiency recognition.

For instance, the solid harmonic scattering transform on a local receptive field had $\left|Q\right|JL+\left|Q\right|JL\left(J-2\right)/2$ invariant coefficients as outputs; we fixed J=7 and q∈Q={1/2,1,2,3} as the principle settings on the solid scattering transform process and set C=128 as the number of local fields. Within each field, we applied a spatial 3D grid with 8×8×8 reference positions, which means we utilized 128×8×8×8 floats as each sample’s density representation; the scattering coefficient representation had a constant dimension in the locality as 84 first-order and 252 second-order coefficients, with 128×336 floats as the overall representation.

(2) The sparse dictionary learning structure is demonstrated in Figure 6. It remained a very wide feature vector when we directly input a batch of scattering coefficients into the ISTC layer; therefore, we applied a 15×1 convolution operation with batch normalization to reduce it to 128×200. Furthermore, it included $3.8\times 10^{5}$ learned parameters. The *N* was set to 3 since it was experimentally sufficient to allow the sparsity to reach the extremum.

The whole framework is illustrated in Figure 6, where the sub-dictionary, $D_{c}$, had 512 atoms, ${\left\{d_{i}\right\}}_{i}$, each with 1×1 support to provide an initial low correlation among the atoms and it met the over-complete condition for each of the locally projected scattering coefficients. This ISTC network took as input an array, LSρ, of size 128×200, and output a sparse code matrix of 128×512. The total number of learned parameters in *D* was about $6.5\times 10^{4}$. The number of parameters in *L* and *D* was around $4.5\times 10^{5}$ in total. Furthermore, in order to generate a classification ability for simulating real human face recognition situations, we adopted a simple linear classifier and evaluated the performance; the results are presented in the following sections.

### 4.3. Hyperparameter Tuning Process

The proposed network was implemented with PyTorch Version 3.7 (Initially Released in September 2016 by Meta AI, Astor Place, New York City, US) [40] and was trained with i7-8700K CPU and a single GTX2080TI GPU.

**Figure 9:**
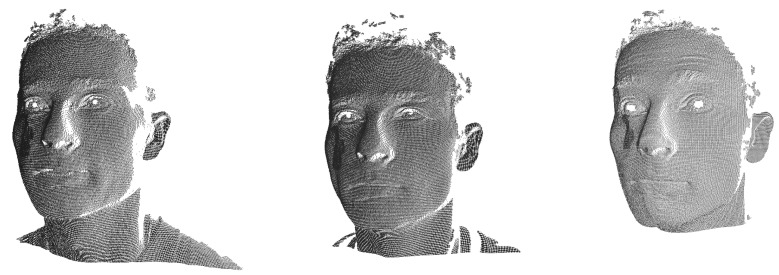
One identity from FRGCv2 with three scans.

We used the Face Recognition Grand Challenge (FRGC) v2.0 dataset and the Bosphorus dataset to evaluate the performance of the proposed face recognition method. It took about 15 hours for our hardware to train the network on each dataset, and our method obtained a high accuracy on FRGCv2 with a short training procedure. Since FRGCv2 has a relatively regular and uniform sampling process—which can be observed in Figure 9 as a general case—it helped to verify our hypothesis. In order to adequately clarify the capability of our framework, we mainly applied the rank-1 recognition rate on the full Bosphorus dataset for the ablation study.

There were three major parameters introduced in the solid harmonic scattering—namely, J, L, and C—which decided the width of the scattering coefficients as the input for the dictionary learning phase; another combination of $dim_{Dictionary}$ and $dim_{LinearProj}$ decided the number of parameters for the dictionary learning. With the experimental observations, the solid harm coefficients had a stepped magnitude distribution along their support, which could be in great disparity, which led to aliasing during the normalization among local patches when we picked insufficient scaling levels, J; however, if we applied a big J, it required excessive computation in return. Given this, we first went through a wide combination space of the above parameters and obtained preliminary results, as presented in Table 1, where we can see a trend in performance improvement in the increasing J, $dim_{Dictionary}$, and $dim_{LinearProj}$. Then, we utilized two-stage training with individual parameter searching, as follows.

**Figure 10:**
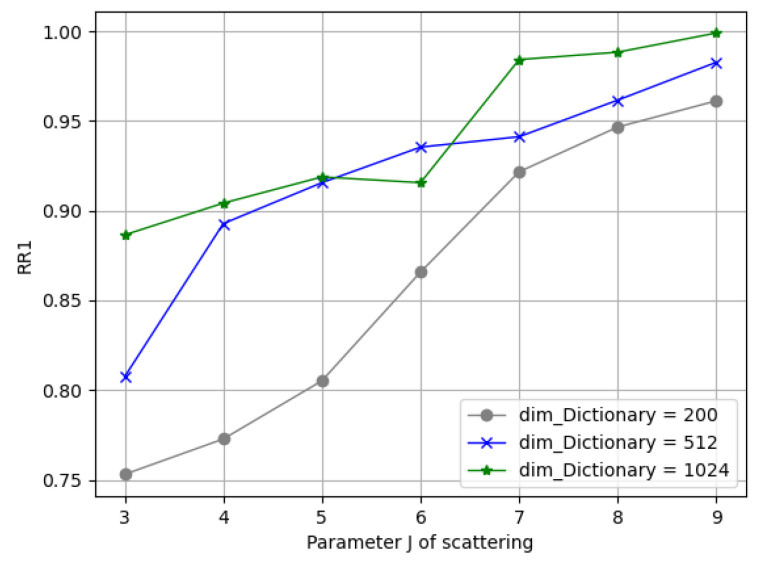
Comparisons of different scattering parameters.

**Figure 11:**
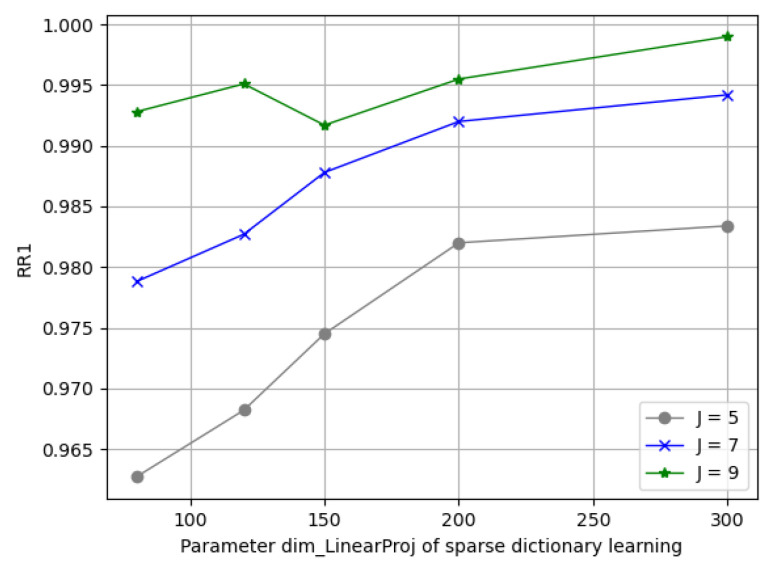
Comparisons of different sparse dictionary learning parameters.

(1) Parameters in Solid Harmonic Scattering: Figure 10 demonstrates the rank-1 recognition rate on the Bosphorus dataset by training the network with J = 3, 4, 5, 6, 7, 8, 9 values under C = 128. It can be seen that the dimension of each sub-dictionary had to be subsequent to satisfy overcompleteness; it can be seen from the blue/green lines that when $dim_{Dictionary}>dim_{scattering}$ and J>6, the recognition rate barely grows.
(2) Parameters in Sparse Dictionary Coding: We fixed $dim_{Dictionary}=512$; here, we found that a variation in J in (5, 7, 9) reached its best spot on $dim_{Linear_{P}roj}⩾150$. Figure 11 depicts the varying performance; we applied [J = 7, $dim_{Linear_{P}roj}$ = 150, *C* = 128, $dim_{Dictionary}$ = 512] as the principle experimental configuration of this framework.

**Table 1:** Rank-1 recognition rate (RR1) under different combinations of (J,L,C) and $\left(\dim_{S},\dim_{L},\dim_{D}\right)$ on Bosphorus dataset.

(J,L,C)	$\left(\dim_{S},\dim_{L},\dim_{D}\right)$	RR1
5,3,128	252,120,512	90.42%
6,3,128	336,120,512	93.54%
7,3,128	432,120,512	88.65%
6,3,128	336,150,512	91.88%
6,3,128	336,200,512	95.42%
7,3,128	336,150,512	95.63%
7,3,128	432,200,512	99.49%

### 4.4. Comparison with Other Methods

(1) Results on the FRGCv2 dataset: The FRGC v2.0 dataset [39] contained 4007 scans of 466 subjects in total; we followed its protocol to train on the Spring2003 partition and used the remaining data for testing. The results of running our proposed method and the state-of-the-art methods on the FRGC v2.0 dataset are shown in Table 2. The methods that used corresponding 2D photos are denoted as (2D+3D) and the ones that used a fine-tuning strategy are marked with (FT). Note that our approach required no information other than the positions of the point clouds; this property allowed for a much simpler sampling process in actual scenarios, whereas the illumination/rotation variants have been “compressed” in our representations. The recognition accuracy of our approach was also competitive with a rank-1 recognition rate of 99.84%.
(2) Results on the Bosphorus dataset: The Bosphorus dataset [38] has 4666 scans collected from 105 subjects, with very rich variants in expression, systematic variations in poses, and different types of occlusions.

**Table 2:** Rank-1 recognition rate (RR1) under FAR = $1\times 10^{-3}$ on FRGC V2.0 dataset.

Method	RR1	VR
Mian et al. [34] (2008)	96.10%	98.60%
Al-Osaimi. [41] (2016)	96.49%	90.00%
Ouamane et al. [42] (2017)	−	96.65%
Ouamane et al. [42] (2017) [2D+3D]	−	98.32%
Gilani and Miancite [3] (2018)	97.06%	−
Gilani and Mian [3] (2018) (FT)	99.88%	−
Cai et al. [1] (2019) (FT)	100.00%	100.00%
Yu et al. [2](2022)	98.85%	96.75%
Ours	99.84%	99.39%

To be specific, almost every subject had varying scans, with 34 expressions; 13 yaw, pitch, and cross rotations; and 4 occlusions (hand, hair, eyeglasses); this dataset was found to be the most convincing experimental context for demonstrating our method’s capacity. We did not exclude any hard samples from this dataset and obtained a 99.90% rank-1 accuracy. The comparison with other methods is illustrated in Table 3.

## 5. Discussion

In general, our method provides an approach to defining a representation that is invariant to isometric transformations up to an induced small scale; additionally, our method enables one to train parameters from a limited number of observations. However, alternative deformations may exist in some inherent behaviors, e.g., aging/expression, which is not isometric. For those kinds of pattern recognition tasks, modifications should be applied to the original structure to capture discriminative features on purpose. The flexibility of this framework has the potential to extend and spread to wider fields, whereas mining geometrical data can play a role in more transparent methodologies.

## 6. Conclusions

This work shows that mere spatial coordination in point cloud faces is sufficient to improve the performance of 3D face recognition beyond the accuracy of other current methods. In addition, the strategy of applying fast-converging sparse dictionary deep learning to select the related features while reducing intraclass variances has created the potential to develop into applications in real time and unbounded real scenarios. Our future research interest is in improving the treatment of point cloud human faces as stochastic processes; we will examine the potential of its application on larger-scale recognition tasks.

## Figures and Tables

**Table 3:** Rank-1 recognition rate (RR1) under FAR = $1\times 10^{-3}$ on Bosphorus dataset.

Method	RR1	VR
Mian et al. [34] (2008)	96.40%	−
Al-Osaimi [41] (2016)	92.41%	93.50%
Lei et al. [31] (2016)	98.90%	−
Ouamane et al. [42] (2017) [2D+3D]	−	96.17%
Gilani and Miancite [3] (2018)	96.18%	−
Gilani and Miancite [3] (2018) (FT)	100%	−
Cai et al. [1] (2019) (FT)	99.75%	98.39%
Yu et al. [2] (2022)	99.33%	97.70%
Ours	99.90%	99.55%

## Data Availability

Not applicable.
